# Supplementary material for: Detection of β-Lactamase-Producing Enterococcus faecalis and Vancomycin-Resistant Enterococcus faecium Isolates in Human Invasive Infections in the Public Hospital of Tandil, Argentina
Source: Pathogens. 2020 Feb 20;9(2):142. doi: 10.3390/pathogens9020142 (PMC7168638; doi:10.3390/pathogens9020142)
Supplement: Supplementary file 1 [file pathogens-09-00142-s001.zip › pathogens-719389-SI.docx]

**Table S1.** Relevant clinical epidemiological data included in this study.

| **Epidemiological data** | ***n* (%)** |
| --- | --- |
| **Type of infection** | **34/55 (61.8)** |
| Prosthetic joint infections | 11 (20.00) |
| Post-traumatic infections | 6 (10.91) |
| Post-surgical infections | 5 (9.10) |
| Intra-abdominal Infections |  |
| *Appendicitis* | 2 (3.64) |
| *Peritonitis* | 2 (3.64) |
| *Vesicular lithiasis* | 1 (1.82) |
| Bacteraemia | 2 (3.64) |
| Sepsis | 1 (1.82) |
| Osteomyelitis | 1 (1.82) |
| Endocarditis | 1 (1.82) |
| Pericarditis | 1 (1.82) |
| Upper urinary tract infection | 1 (1.82) |
| **Clinical samples** | ***n* = 55** |
| Blood | 22 (40.00) |
| Abdominal fluid | 6 (10.91) |
| Peritoneal fluid | 6 (10.91) |
| Intravesicular fluid | 1 (1.82) |
| Abscess fluid | 12 (21.82) |
| Synovial fluid | 7 (12.73) |
| Pericardial fluid | 1 (1.82) |
| **Baseline disease** | **28/55 (50.9)** |
| DBT* type I | 5 (9.09) |
| DBT* type II | 1 (1.82) |
| Chronic renal insufficiency | 4 (7.27) |
| Rheumatoid arthritis | 1 (1.82) |
| Alcoholism | 3 (5.45) |
| Osteoarthritis | 1 (1.82) |
| Cancer | 8 (14.55) |
| ACV | 1 (1.82) |
| EIP | 1 (1.82) |
| HIV | 1 (1.82) |
| Lithiasis | 1 (1.82) |
| Without pathology | 1 (1.82) |
| **Gender** | ***n*=55** |
| Female | 15 (27.27) |
| Male | 40 (72.73) |
| **Polymicrobial samples** | **7/55 (12.73)** |
| **Previous antibiotic therapy** | **48/55 (87.27)** |
| **Evolution** |  |
| Fatal cases | 15 (27.27) |
| Medical discharge | 40 (72.73) |

Abbreviations: DBT, diabetes mellitus; ACV, cerebrovascular accident; EIP, Pelvic Inflammatory Disease (Health Sciences Descriptors), HIV, Human immunodeficiency virus.
